# Association of plasma endothelial lipase levels on cognitive impairment

**DOI:** 10.1186/s12888-019-2174-8

**Published:** 2019-06-19

**Authors:** Sang-Moon Yun, Jee-Yun Park, Sang Won Seo, Jihyun Song

**Affiliations:** 1 Division of Brain Diseases, Center for Biomedical Sciences, Korea National Institute of Health, Cheongju-si, Chungcheongbuk-do 28159 Republic of Korea; 2 Department of Neurology, Samsung Medical Center, Sungkyunkwan University School of Medicine, Seoul, 06351 Republic of Korea

**Keywords:** Alzheimer’s diseases (AD), Endothelial lipase (EL), Clinical dementia rating (CDR), Cognitive impairment, High density lipoprotein cholesterol (HDL-C)

## Abstract

**Background:**

Peripheral high-density lipoprotein cholesterol (HDL-C) has been known to influx into the brain and be inversely associated with the risk of Alzheimer’s disease (AD). However, recent prospective studies of the association between HDL-C and AD have yielded inconsistent results. Here, we examined the association between the endothelial lipase (EL), which is known to be major determinant of HDL-C levels, and cognitive function.

**Method:**

We compared plasma from 20 patients with Alzheimer’s disease (AD), 38 persons with mild cognitive impairment, and 51 cognitively normal controls. Plasma EL levels were measured using the enzyme-linked immunosorbent assay.

**Results:**

EL levels were inversely correlated with HDL-C, as previously reported; however, there were no mean differences in plasma EL between the diagnostic groups. An analysis by classification of dementia severity according to clinical dementia rating (CDR) showed that the EL levels were significantly higher in the CDR1 group (mild dementia), as compared to CDR0 (no dementia), CDR0.5 (very mild), and CDR2 (moderate) groups. Prior to moderate dementia stage, trends analysis showed that EL levels tended to increase with increasing severity (*p* for trend = 0.013). Consistently, elevated EL levels were significantly correlated with the mini-mental state examination (MMSE) score (r = − 0.29, *p* = 0.003). Logistic regression for association between plasma EL and cognitive impairment (MMSE score ≤ 25) showed that participants with EL levels in the upper range (> 31.6 ng/ml) have a higher adjusted odds ratio of cognitive impairment than those within the lower EL range.

**Conclusion:**

Findings from the present study reflect the association of EL and cognition, suggesting that the individuals with elevated plasma EL concentration are at an increased risk of cognitive impairment.

**Electronic supplementary material:**

The online version of this article (10.1186/s12888-019-2174-8) contains supplementary material, which is available to authorized users.

## Background

Lipid metabolism has a potential role in the development and progress of Alzheimer’s disease (AD). Abnormal lipid in the brain has been considered as a histological feature of AD [1]. The Apolipoprotein E (ApoE) gene is known to play a role in neuronal lipid homeostasis in the brain, and ApoE genotype has been associated with an increased risk of AD [2]. Genome-wide association studies have shown that lipid metabolism and transport are one of the main pathways involved in the pathological process of AD [3, 4].

Furthermore, autopsy studies have shown that an abnormal lipid profile in peripheral blood precedes the pathological characteristics of AD [5–7]. A recent meta-analysis of cohort studies found that increased cholesterol in mid-life, not in late-life, is associated with incident dementia in old age [8]. This current evidence supports an involvement of dyslipidemia in development of AD.

Recent prospective studies with moderate to large sample sizes have suggested that high- density lipoprotein cholesterol (HDL-C), one of the blood lipid parameters, may be inversely associated with the risk of AD [9–11]. However, the relatively small epidemiological studies that have subsequently investigated the association between serum HDL-C and AD risk have yielded inconsistent results [12, 13]. Inconsistent findings for a link between HDL-C and AD risk may come from misclassification of dementia resulting from varying diagnostic criteria [11] and insufficient follow-up times to show the effect of HDL-C on incident AD [8]. In addition, HDL-C measurement is relatively less standardized and less precision, compared with total cholesterol (TC) [14]. Because clinically meaningful differences in concentrations are small, the HDL-C measurement error might contribute to these conflicting results [15].

The endothelial lipase (EL, also alternatively named LIPG) plays an important role in HDL metabolism. EL hydrolyzes HDL phospholipids and clears HDL-C from the circulation [16, 17]. Gain and loss of function studies in mice have indicated that EL is major determinant of HDL-C [18–20]. Genetic variation studies have supported a positive correlation between EL and blood HDL-C in humans [21, 22]. EL does not affect other lipid-related blood parameters [23]. EL is secreted by vascular endothelial cells, medial smooth muscle, and macrophages on atherosclerotic lesions [24]. Induced inflammation in endothelial cells treated with TNF-α or interleukin-1β as well as in mice on LPS administration results in elevation levels of EL mRNA and protein [25–27]. These results suggest that EL expression is regulated by inflammatory stimuli.

So far, evidence for the role of HDL-C on cognitive decline and dementia, and that for the role of EL on determining of HDL-C has been separately accumulated; the link between EL and cognition has not yet been studied. Thus, in the current study, we examined whether the blood EL concentrations were associated with cognitive impairment in a cross-sectional study of elderly people in Korea.

## Methods

### Subjects

We recruited 97 participants including cognitively normal individuals (*N* = 39), amnestic MCI (*N* = 38), and AD-type dementia (*N* = 20) from the memory clinic in Samsung Medical Center, from February 2017 to August 2017. Additionally, we recruited 29 cognitively normal participants from the local community. Five participants were excluded due to the withdrawal of consent, and a participant was excluded due to the failure of obtaining an amyloid PET scan. Of the remaining 120 participants, 11 subjects with a coefficient of variation of 30% or more in the EL measurements were excluded. A total of 109 elderly participants (mean age; 75.3 years) over 65 years of age were recruited from a clinic and local community (Additional file 1: Figure S1). The subjects consisted of 51 cognitively normal controls (NC), 38 patients with amnestic mild cognitive impairment (aMCI), and 20 patients with AD. All participants underwent neurological examination, neuropsychological (NP) testing, and routine blood tests. Blood tests for all participants included a complete blood count, blood chemistry tests, vitamin B12/folate, syphilis serology, thyroid function tests and APOE genotyping. Participants with current or past neurological or psychiatric illnesses such as major depressive disorders, epilepsy, brain tumors, encephalitis or severe head trauma that would affect cognitive function were excluded. On MRI, patients with structural lesions such as tumors, traumatic brain injuries, or hydrocephalus were also excluded. The institutional review boards approved this study. Written, informed consent was obtained from patients and caregivers.

The NC was volunteered community based elderly who had no history of neurological or psychiatric illness, and had no abnormalities upon neurological examination. The NC exhibited normal cognition on the detailed neuropsychological tests. Patients met the following criteria for aMCI proposed by Petersen et al. [28]: (1) subjective memory complaints by the patient or an informant; (2) relatively normal performance in other cognitive domains; (3) normal activities of daily living (ADL), as judged clinically; (4) objective memory decline below − 1.0 SD on either verbal or visual memory tests; and (5) not demented. AD was diagnosed based on National Institute on Aging-Alzheimer’s Association (NIA-AA) research criteria for probable AD [29].

### Neuropsychological tests

All participants underwent a standardized neuropsychological battery called the Seoul Neuropsychological Screening Battery, which consisted of tests of attention, language, visuospatial, memory, and frontal/executive functions [30]. Tests that were scored included the following: The Korean version of the Boston Naming Test (K-BNT), Rey-Osterrieth Complex Figure Test (RCFT: copying, immediate and 20-min delayed recall, and recognition), Seoul Verbal Learning Test (SVLT: immediate, 20-min delayed recall and recognition), phonemic and semantic Controlled Oral Word Association Test (COWAT) and the Stroop Test (word and color reading). SVLT and RCFT were performed to assess verbal and nonverbal learning and memory. The K-BNT and RCFT copy were done to evaluate language and visuospatial function, respectively. Phonemic and semantic COWAT and the Stroop Test were performed to evaluate frontal/executive function. Scores were considered abnormal when they were lower than − 1.0 SD below age- and education-adjusted norms. All participants also were administered the Clinical Dementia Rating Scale Sum of Boxes (CDR-SOB). Clinical Dementia Rating (CDR) on 5-point scale was scored using the CDR-SOB. The Korean version of the Mini-Mental Status Examination (K-MMSE, range 0 to 30) was also administered. Tests were administered by experienced staff and supervised by board-certified clinical neuropsychologists.

### Amyloid PET data acquisition and positivity

Patients underwent florbetaben (FBB) PET or flutemetamol (FMM) PET at Samsung Medical Center using a Discovery STe PET/CT scanner (GE Medical Systems, Milwaukee, WI, USA) in three-dimensional scanning mode. ^18^F-florbetaben PET was classified as positive when visual assessment was scored as 2 or 3 on brain amyloid-plaque load (BAPL) scoring system [31, 32]. Visual interpretation of ^18^F-flutemetamol PET images relied upon a systematic review of five brain regions (frontal, parietal, posterior cingulate and precuneus, striatum and lateral temporal lobes) [33]. More detailed procedures are described in supplementary methods section (Additional file 1: Methods).

### Measurement of plasma EL

The plasma concentrations of EL were measured using the ELISA kit (Cloud-Clone Corp., Houston, TX; SEA469Hu) according to the manufacture’s protocol. Plasma sample was separated with EDTA as anticoagulant. EL release is induced by administration of heparin, but EL levels in pre heparin condition are significantly correlated with levels of post heparin condition [34]. To assess the correlation between plasma EL mass and cognitive impairment, therefore, EDTA-plasma was obtained without administration of heparin. Each level is mean values of the duplicate measurement.

### Statistical analysis

Group differences were calculated using either t-tests or an analysis of variance (ANOVA) followed by post hoc tests for continuous variables and chi-square tests for nominal variables. Linear trend was estimated using the ANOVA results and with a polynomial contrast. Pearson correlation coefficients were used to quantify associations between variables. Simple and multiple logistic regression analyses were conducted to calculate odds ratios (ORs) of cognitive impairment (MMSE score ≤ 25) per 1 standard deviation (SD) increment of plasma EL concentration. The MMSE cut-off chosen for the general cognition function varied from 23 to 26 [35]. We determined MMSE score ≤ 25 as cognitive impairment. All calculations, graphs and statistical analyses were performed using SPSS ver. 19.0 (IBM Corporation, Armonk, NY) and GraphPad Prism 5 (GraphPad Inc., La Jolla, CA).

## Results

### Demographic and clinical data

Demographic and clinical data are shown in Table 1. The mean age of patients with AD was 79.1 years old, which was significantly older than the cognitively normal control (NC) group (73.8 years old*, p* <  0.001) and MCI group (75.3 years old*, p* <  0.05). There was no between-group difference in the number of years of education, body mass index (BMI) score or lipid parameters, such as TC, HDL-C, low-density lipoprotein cholesterol (LDL-C) and triglyceride (TG). But between-group difference in sex ratios, total protein or albumin was observed. ApoE4 allele carriers in the AD group comprised 55%, which was significantly more frequent than those in the NC (22%) and MCI (37%) groups. Positive Amyloid PET brain scans were found in 18% of the control group, 47% of the MCI group and 65% of the AD group. The average MMSE score of the MCI group (25.9 ± 2.7) was lower than control group (27.6 ± 2.1; *p* <  0.05), and the average MMSE score of the AD group (16.6 ± 6.4) was lower than both other groups (both *p* <  0.001). Clinical dementia rating (CDR) scores were higher in AD group compared to both other groups (both, *p* <  0.001). However, the difference in CDR score between NC and MCI groups was not observed.

**Table 1:** Participant demographics, clinical data, blood tests, and EL concentration according to diagnosis

	Total	control	MCI	AD	*P-value*
N	109	51	38	20	
Age	75.3 ± 5.7	73.8 ± 4.9	75.3 ± 5.0	79.1 ± 7.2	0.002
Sex, male/female N (female %)	37/72 (66%)	10/41 (80%)	19/19 (50%)	8/12 (60%)	0.009*
Education, years	9.8 ± 4.7	9.4 ± 5.2	10.6 ± 3.9	9.2 ± 4.9	0.413
BMI	24.0 ± 2.7	24.2 ± 2.6	23.8 ± 2.7	24.0 ± 2.9	0.829
Total Protein (g/dL)	7.0 ± 0.5	7.1 ± 0.5	7.0 ± 0.4	6.8 ± 0.5	0.046
Albumin (g/dL)	4.3 ± 0.3	4.4 ± 0.3	4.4 ± 0.3	4.2 ± 0.3	0.010
Total Cholesterol (mg/dL)	177.0 ± 32.0	174.1 ± 30.2	175.8 ± 31.4	186.8 ± 37.3	0.312
HDL-C (mg/dL)	54.0 ± 16.5	56.9 ± 16.7	53.2 ± 16.4	50.1 ± 16.4	0.365
LDL-C (mg/dL)	108.9 ± 30.0	104.9 ± 30.2	109.3 ± 28.6	118.2 ± 31.3	0.246
Triglyceride (mg/dL)	149.3 ± 84.6	138.5 ± 62.4	152.0 ± 95.8	171.4 ± 108.2	0.330
ApoE4 carrier, N (%)	36 (33%)	11 (22%)	14 (37%)	11 (55%)	0.022*
Amyloid PET positive N (%)	40 (37%)	9 (18%)	18 (47%)	13 (65%)	< 0.001*
CDR	0.6 ± 0.5	0.4 ± 0.2	0.5 ± 0.1	1.5 ± 0.8	< 0.001*
CDR-SOB	2.4 ± 3.7	0.7 ± 0.4	1.5 ± 1.1	8.8 ± 4.7	< 0.001
MMSE	25.0 ± 5.3	27.6 ± 2.1	25.9 ± 2.7	16.6 ± 6.4	< 0.001
EL (ng/ml)	21.4 ± 10.1	21.0 ± 8.8	21.9 ± 9.7	21.7 ± 14.2	0.899

The continuous value is represented by mean ± SD

*p*-values are for the analysis of variance (ANOVA), * for chi-squared test of independence

Abbreviation: *BMI* body mass index, *HDL-C* high density lipoprotein cholesterol, *LDL-C* low-density lipoprotein cholesterol, *ApoE*, Apolipoprotein E; CDR, clinical dementia rating; CDR-SOB, clinical dementia rating scale sum of boxes, *MMSE* mini-mental state examination, *EL* endothelial lipase

### Relationships of EL with cognitive impairment

Plasma EL showed no significant between-diagnostic group differences (Table 1). EL was inversely correlated with HDL-C (r = − 0.24, *p* = 0.015) in the blood lipid profile, and was not correlated with TC, LDL-C, or triglyceride (Additional file 1: Figure S2). Additionally, plasma EL was positively correlated with white blood cell (WBC) and platelet counts. (Additional file 1: Table S1).

**Fig. 1:**
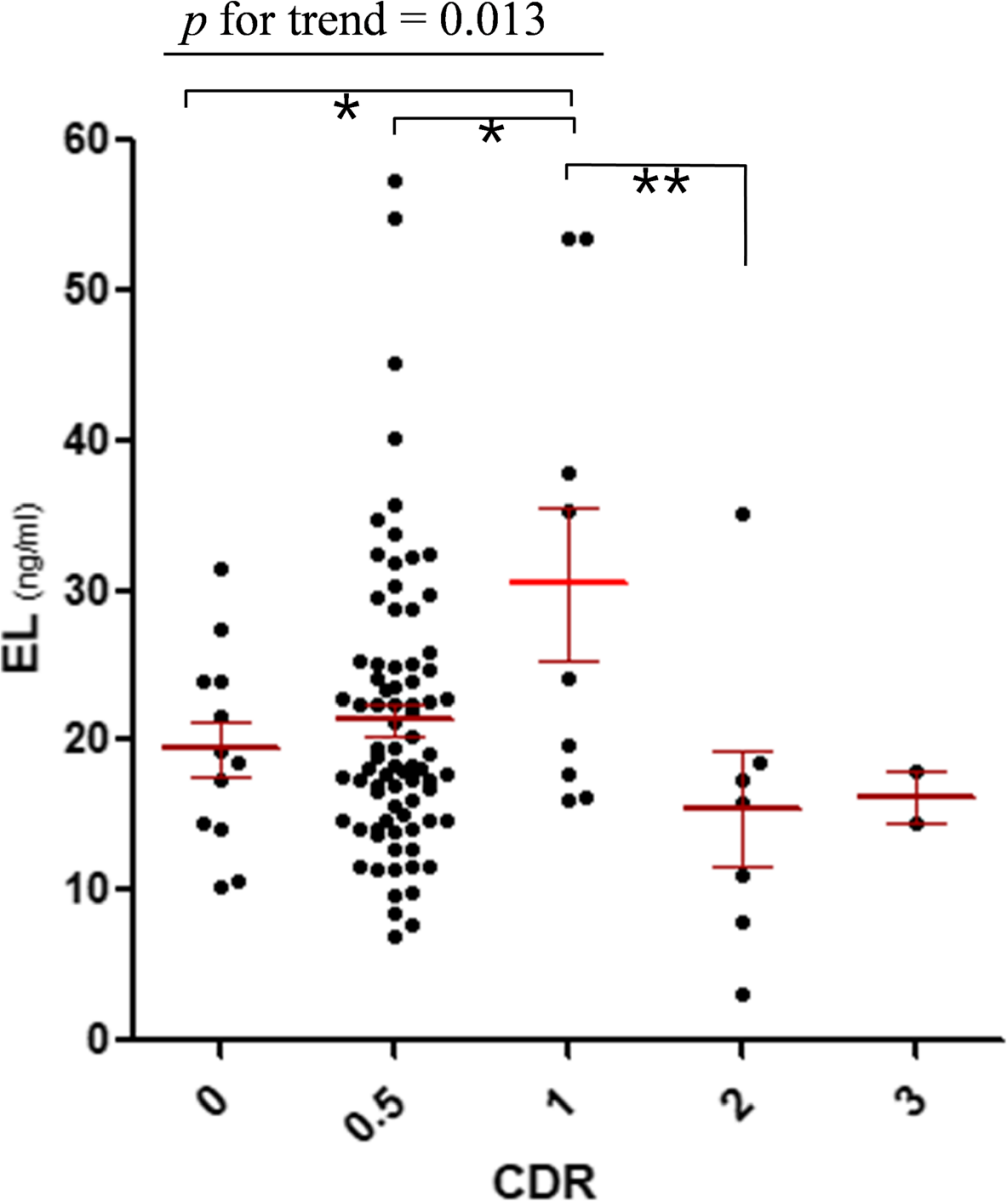
Plasma EL levels according to CDR scores. The grouped Scatter plot shows plasma EL concentrations across CDR scores. Each level is mean values of the duplicate measurement, below the coefficient of variation < 30%. Red bars represent mean ± S.E.M of each CDR group. Comparisons were made using a One-way ANOVA, followed by post-hoc analysis with the Least Significant Difference test. Significance is indicated with * and ** representing *p* <  0.05 and *p* <  0.01 respectively. The linear trend was analyzed from CDR0 to CDR1. Abbreviations: CDR, clinical dementia rating; EL, endothelial lipase

The analysis by classification of dementia severity according to CDR scores, showed that there were significant group differences between CDR groups (Fig. 1; one-way ANOVA; *p* = 0.03). The EL levels were significantly higher in the CDR 1 group (30.5 ± 5.1) than in the CDR 0 (19.4 ± 1.9, *p* = 0.012), CDR 0.5 (21.4 ± 1.1, *p* = 0.01) or CDR 2 (15.5 ± 3.9, *p* = 0.003) groups (Fig. 1). There was no trend of EL concentration in the whole CDR severity range. However, as the severity of dementia increased from CDR = 0 to CDR = 1, the EL concentration tended to increase (*p* for trend = 0.013; Fig. 1) and elevated EL levels were significantly correlated with MMSE score (r = − 0.29, *p* = 0.003; Additional file 1: Figure S3). However, the elevated levels of plasma EL were not correlated with the presence of ApoE4 (*p* = 0.265) or amyloid deposit in the brain (t-test *p* = 0.199) (Additional file 1: Figure S4).

To determine the association between EL and cognitive impairment (MMSE score ≤ 25), we investigated ORs in subjects from cognitively normal to mild dementia (Table 2 and Table 3). EL concentration was categorized into two ranges according to the mean + 1 standard deviation (31.6 ng/ml). Participants that fell into the upper range of EL had a higher unadjusted OR of cognitive impairment (OR = 5.4, 95% confidence interval [CI], 1.7–17.5) than those that fell into lower range. When adjusted for age, sex, education, BMI, ApoE e4 allele and Amyloid-PET imaging, and additionally adjusted for disease history including diabetes hyperlipidemia, heart disease, hypertension, stoke, traumatic brain injury, the ORs of cognitive impairment in the upper range group compared with the lower range group were similar to those of the unadjusted model (OR = 4.8, 95% CI 1.3–17.9; OR = 5.6, 95% CI 1.4–22.9, respectively)..

**Table 2:** Participant demographics, clinical data, blood tests, and history of disease according to plasma EL concentration prior to moderate dementia stage

	Categorization into the mean + 1 SD of plasma EL concentration			
	Lower range of EL	Upper range of EL	Total	*p-value*
	< 31.6 ng/ml, *n* = 85	≥ 31.6 ng/ml, *n* = 15	*n* = 100	
EL (ng/ml)	18.6 ± 5.6	40.7 ± 9.5	22.1 ± 10.1	< 0.001
Age	74.5 ± 5.3	77.9 ± 5.3	75.0 ± 5.4	0.025
Sex, male/female N (female %)	56 (66%)	9 (60%)	65 (65%)	0.66 *
Education, years	9.8 ± 4.6	9.0 ± 5.4	9.7 ± 4.7	0.589
BMI	24.1 ± 2.7	23.6 ± 1.9	24.0 ± 2.6	0.486
Total Protein (g/dL)	7.0 ± 0.5	7.0 ± 0.5	7.0 ± 0.5	0.878
Albumin (g/dL)	4.4 ± 0.3	4.3 ± 0.3	4.4 ± 0.3	0.635
ApoE4 carrier, N (%)	24 (28%)	6 (40%)	30 (30%)	0.359 *
Amyloid-PET positive N (%)	27 (32%)	6 (40%)	33 (33%)	0.532 *
CDR	0.5 ± 0.2	0.6 ± 0.2	0.5 ± 0.2	0.006 *
CDR-SOB	1.3 ± 1.4	2.8 ± 3.0	1.5 ± 1.8	0.074
MMSE	26.5 ± 3.3	22.6 ± 5.5	25.9 ± 3.6	0.017
Diagnosis				
Control, N	47	4	51	0.042 *
MCI, N	31	7	38	
AD, N	7	4	11	
Disease history, N(%)				
Diabetes	12 (14%)	3 (20%)	15 (15%)	0.556 *
Hypertension	43 (51%)	8 (53%)	51 (51%)	0.845 *
Hyperlipidemia	40 (47%)	7 (47%)	47 (47%)	0.978 *
Heart disease	10 (12%)	2 (13%)	12 (12%)	0.863 *
Stroke	4 (5%)	0 (0%)	4 (4%)	0.391 *
Traumatic brain injury	4 (5%)	0 (0%)	4 (4%)	0.391 *

The continuous value is represented by mean ± SD

*p*-values are for the t-test, * for chi-squared test of independence

Abbreviation: *BMI* body mass index, *ApoE* Apolipoprotein E, *CDR* clinical dementia rating, *CDR-SOB* clinical dementia rating scale sum of boxes, *MMSE* mini-mental state examination, *EL* endothelial lipase

**Table 3:** ORs for cognitive impairment according to plasma EL concentration prior to moderate dementia stage

	Categorization into the mean + 1 SD of plasma EL concentration		
	Lower range of EL	Upper range of EL	*p* value
	< 31.6 ng/ml, *n* = 85	≥ 31.6 ng/ml, *n* = 15	
Not adjusted	1 (ref.)	5.4 (1.7–17.5)	0.005
Adjusted for covariates^a^	1 (ref.)	4.8 (1.3–17.9)	0.021
Adjusted for covariates^b^	1 (ref.)	5.6 (1.4–22.9)	0.016

Figures in brackets after each odds ratio indicated 95% confidence intervals

^a^Age, sex, education, BMI, APOE-e4 allele, Amyloid PET

^b^Age, sex, education, BMI, APOE e4 allele, Amyloid PET, history of disease (diabetes, hyperlipidemia, heart disease, hypertension, stroke, traumatic brain injury)

## Discussion

In the present study, we demonstrated that plasma EL concentration was associated with cognitive impairment in a sample of elderly Korean people. First, we assessed that there was no mean difference in EL levels between diagnostic groups of dementia, MCI and NC. Secondly, we used CDR, which represent dementia severity, to compare the differences in EL levels between the degree of cognitive impairment. Levels of plasma EL were significantly higher in the CDR1 group compared to both the less severe stages (CDR0 and CDR0.5) and the more severe stage (CDR2). Prior to the CDR2 stage, EL levels had a tendency to increase with increasing severity of dementia. Thirdly, we used the MMSE score to reconfirm the relevance between cognitive impairment and EL. Elevated EL levels were significantly associated with reduction of cognitive function.

This is the first study to determine the association between EL and cognitive function. Elevated EL levels in individuals under CDR1 were significantly correlated with cognitive impairment, as assessed by the MMSE. Moreover, logistic regression analysis of the association between upper EL (> 31.6) and cognitive impairment (MMSE score ≤ 25) showed that participants with an upper EL range had at a higher risk (adjusted Odds Ratio = 5.6; *p*-value = 0.016) of cognitive impairment than those with a lower range. Recently, a relevant study investigated the effect of EL common variant on AD [36]. The EL variant carrier suggested showing at a higher risk of AD.

EL facilitates the hydrolysis of HDL phospholipids and clears HDL-C from the circulation [16]. EL is known as a major regulator of HDL-C and does not affect other lipid parameters [20, 21, 23]. High HDL-C has been associated with better memory performance, while low HDL-C has been associated with a decline in memory and cognition [37–39]. Consistent with this work, we observed higher HDL-C levels when there were lower EL levels (Additional file 1: Fig. S2). However, other lipid profiles, such as TC, LDL-C, and triglyceride (TG), were not correlated with EL. Notably, there was a significant difference in EL levels between CDR groups (Fig. 1), but no significant differences in HDL-C levels between CDR groups (*p* = 0.85, Additional file 1: Table S2). These results suggest that EL concentration may better reflect the severity of dementia than HDL-C levels.

Inflammation which is a necessary and adaptive defense response to different harmful stimuli has been linked to dementia [40–42]. Systemic and chronic inflammation in which immune system is over-activated, can lead to an attack on healthy brain cells and the subsequent progression to dementia [43, 44]. Infectious pathogens, such as fungus [45, 46], bacteria [47], viruses [48] can directly and indirectly induce neuro-inflammation, leading to AD pathology [49]. Consistent with these findings, we observed that EL was correlated with peripheral platelet and white blood cell counts, which are blood inflammatory markers [50, 51]. These results are consistent with evidence that EL levels are positively correlated with other inflammatory markers, C-reactive protein and interleukin 6 [52–54], and that its mRNA and protein levels are regulated by cytokine and LPS [25–27].

We found that EL levels tended to increase with dementia severity prior to the CDR2 stage, but decreased at CDR2 and CDR3 stages. This pattern, which shows the highest peak in the middle of disease progression, is similar to the previous results of MCP-1 and sTREM2 studies [55, 56]. Higher levels of inflammation have observed in earlier stages of the dementia, suggesting that inflammation precedes development of dementia. [55, 57]. Therefore, the likely reason for highest pattern is that EL levels may be relevant in inflammation. On the other hand, EL levels in the late AD stage might be an effect of drug treatment for conditions such as AD and other concomitant disorders such as hypercholesterolemia. Statins cause a decrease in the expression of EL as well as an increase in HDL-C [58, 59]. Additionally, because of the pro-inflammatory effect resulting in EL expression, anti inflammatory drugs may induce EL inactivation [60]. Most patients with AD have a comorbidity, which can include hypertension (20–30%), being overweight or obese (20–40%), diabetes (20–25%), hypercholesterolemia (> 40%), anemia (> 20%), or cerebrovascular damage (60%) [61]; comorbidities require the administration of multiple drugs concurrently. This polypharmacy might lead to lower EL concentrations in patients with severe AD.

This study has some limitations. First, the average participant age was so high (average 75.8 years old) that even the NC group was likely to have slight cognitive loss. In fact, some participants felt subjective memory impairment. Indeed, there were no significant differences in CDR and CDR-SOB scores between the MCI (CDR 0.5 ± 0.1, CDR-SOB 1.5 ± 1.1) and NC (CDR 0.4 ± 0.2, CDR-SOB 0.7 ± 0.4) groups, although the CDR and CDR-SOB scores were greater in patients with AD (CDR 1.5 ± 0.8, CDR-SOB 8.8 ± 4.7). This may have resulted in the absence of any significant differences in the mean EL concentrations between diagnostic groups. Second, this study did not completely exclude the effect of drugs on EL levels. When we adjusted for disease history, significance of the adjusted OR and 95% CI of cognitive impairment in the upper EL range was maintained. Nonetheless, the information of disease history was derived from questionnaires and might lead to inaccuracies due to errors in subjective memory. Third, this was a single center study. Small and single center study may be in implicit bias regarding to ethnicity. The samples might not be representative, because we performed continuously rather than random sampling. Other center or multi-center validation studies are needed to address sampling errors and limitation of single center study. This was also a cross sectional study, and thus, the causal relationship between EL and cognitive impairment could not be determined. Further prospective and retrospective studies are required to assess the risk factors of EL on cognitive decline.

## Conclusions

Despite evidence of HDL-C association with cognitive function and evidence of EL role as an HDL-C modulator, EL relevance in cognition was not studied. Here, we found to link plasma EL to cognitive impairment. We showed that plasma EL protein tends to increase with cognitive impairment, from cognitively normal to mild dementia cases. Elevated blood EL was associated with an increased risk of cognitive impairment. These results suggest that EL levels are likely to be relevant in the dementia when the process of change is underway rather than when the moderate damage has occurred. Further research should confirm the relevance between EL and cognition in a large population and in a prospective cohort for validation as cognition impairment and its progression biomarker.

## Additional file

Additional file 1: **Table S1.** Correlation between biomarker and plasma EL. **Table S2.** Participant demographics, clinical data, blood test, and history of disease according to plasma EL concentration in advance stage of moderate dementia. **Figure S1.** Flow chart of the recruitment processes. **Figure S2.** Comparison between blood lipid profile and plasma EL. **Figure S3.** The correlation between EL and cognitive impairment in cognitively normal to mild dementia cases. **Figure S4.** The relationship between plasma EL and amyloid pathology or APOE4. (DOCX 623 kb)

## Data Availability

The data analysed during this study are available from the corresponding author on reasonable request. They are also available on request from the national biobank of Korea.

## Abbreviations

AD: Alzheimer’s Diseases
ANOVA: Analysis of variance analysis
ApoE: Apolipoprotein E
BMI: Body mass index
CDR: Clinical dementia rating
CDR-SOB: Clinical Dementia Rating Scale Sum of Boxes
EL: Endothelial lipase
Hb: Hemoglobin
HDL-C: High density lipoprotein cholesterol
LDL-C: Low-density lipoprotein cholesterol
MCI: Mild cognitive impairment
MMSE: Mini-mental state examination
NC: Cognitively normal controls
OR: Odds ratio
RBC: Red blood cell
TC: Total cholesterol
TG: Triglyceride
WBC: White blood cell
