# Supplementary material for: Association of plasma endothelial lipase levels on cognitive impairment
Source: BMC Psychiatry. 2019 Jun 19;19:187. doi: 10.1186/s12888-019-2174-8 (PMC6585097; doi:10.1186/s12888-019-2174-8)
Supplement: Supplementary file 1 — Table S1. Correlation between biomarker and plasma EL. Table S2. Participant demographics, clinical data, blood test, and history of disease according to plasma EL concentration in advance stage of moderate dementia. Figure S1. Flow chart of the recruitment processes. Figure S2. Comparison between blood lipid profile and plasma EL. Figure S3. The correlation between EL and cognitive impairment in cognitively normal to mild dementia cases. Figure S4. The relationship between plasma EL and amyloid pathology or APOE4. (DOCX 623 kb) [file 12888_2019_2174_MOESM1_ESM.docx]

Additional file 1: Supplementary data

**Association of plasma endothelial lipase levels on cognitive impairment**

Sang-MoonYun, Jee-Yun Park, Sang Won Seo, and Jihyun Song

**Supplementary Methods**

**MRI data acquisition**

We acquired MRI data from all participants at Samsung Medical Center, using the same 3.0 T MRI scanner (Philips Achieva; Philips Healthcare, Andover, MA).

**Amyloid PET data acquisition**

Patients underwent florbetaben (FBB) PET or flutemetamol (FMM) PET at Samsung Medical Center using a Discovery STe PET/CT scanner (GE Medical Systems, Milwaukee, WI, USA) in three-dimensional scanning mode that examined 47 slices of 3.3mm thickness spanning the entire brain. CT images were acquired using a 16-slice helical CT (140 KeV, 80 mA; 3.75 mm section width) for attenuation correction. For FBB PET and FMM PET, a 20-minute emission PET scan with dynamic mode (consisting of 4×5 min frames) was performed 90 min after injection of a mean dose of 311.5 MBq FBB and 185 MBq FMM, respectively. Three dimensional PET images were reconstructed in a 128×128×48 matrix with 2×2×3.27mm voxel size using the ordered-subsets expectation maximization (OSEM) algorithm (FBB iteration = 4 and subset = 20; FMM, iteration = 4 and subset = 20).

**Amyloid PET positivity**

All PET images were reviewed by nuclear medicine physicians who were blinded to Neuropsychological testing and classification and dichotomized as amyloid positive or negative using visual reads. ^18^F-florbetaben PET was classified as positive when visual assessment was scored as 2 or 3 on brain amyloid-plaque load (BAPL) scoring system [[1](#_ENREF_1), [2](#_ENREF_2)]. Specifically, interpreters used a regional cortical tracer uptake (RCTU) scoring system (RCTU 1, no tracer uptake; RCTU 2, moderate tracer uptake; and RCTU 3, pronounced tracer uptake) in four brain areas (lateral temporal cortex, frontal cortex, posterior cingulate cortex/precuneus, and parietal cortex). An RCTU score of 1 in each brain region corresponded to a BAPL score of 1, and an RCTU score of 2 in any brain region and no score 3 corresponded to a BAPL score of 2. An RCTU score of 3 in any of the four brain regions corresponded to a BAPL score of 3. BAPL score 1 was interpreted as Aβ - and BAPL score 2 or 3 was interpreted as Aβ +. Visual interpretation of ^18^F-flutemetamol PET images relied upon a systematic review of five brain regions (frontal, parietal, posterior cingulate and precuneus, striatum and lateral temporal lobes) [[3](#_ENREF_3), [4](#_ENREF_4)]. If any one of the brain regions systematically reviewed for ^18^F-flutemetamol PET was positive in either hemisphere, the scan was considered positive [[3](#_ENREF_3)]. Positive scans had one or more regions with increased cortical grey matter signal (above 50-60% peak intensity) and/or reduced (or absent) grey-white matter contrast (white matter sulcal pattern is less distinct) [[5](#_ENREF_5)]. Otherwise, the scan was considered negative.

**Supplementary Reference**

1. Barthel H, Gertz HJ, Dresel S, Peters O, Bartenstein P, Buerger K, Hiemeyer F, Wittemer-Rump SM, Seibyl J, Reininger C *et al*: **Cerebral amyloid-beta PET with florbetaben (18F) in patients with Alzheimer's disease and healthy controls: a multicentre phase 2 diagnostic study**. *Lancet Neurol* 2011, **10**(5):424-435.

2. Kim HJ, Cho H, Werring DJ, Jang YK, Kim YJ, Lee JS, Lee J, Jun S, Park S, Ryu YH *et al*: **18F-AV-1451 PET Imaging in Three Patients with Probable Cerebral Amyloid Angiopathy**. *Journal of Alzheimer's disease : JAD* 2017, **57**(3):711-716.

3. Farrar G: **Regional Visual Read Inspection of [18F]flutemetamol Brain Images from End-of-Life and Amnestic MCI subjects**. *Journal of nuclear medicine : official publication, Society of Nuclear Medicine* 2017, **58**

4. G. F: **Regional Visual Read Inspection of [18F]flutemetamol Brain Images from End-of-Life and Amnestic MCI subjects**. *Journal of nuclear medicine : official publication, Society of Nuclear Medicine* 2017, **58**

5. Gabriel Martínez, Leon Flicker, Robin WM Vernooij, Paulina Fuentes Padilla, Javier Zamora, Marta Roqué i Figuls, Gerard Urrútia, Cosp XB: **18F PET ligands for the early diagnosis of Alzheimer’s disease dementia and other dementias in people with mild cognitive impairment (MCI)**. *The Cochrane database of systematic reviews* 2016.

**List of tables and figures**

**Table S1.** Correlation between biomarker and plasma EL

**Table S2.** Participant demographics, clinical data, blood test, and history of disease according to plasma EL concentration in advance stage of moderate dementia

**Figure S1.** Flow chart of the recruitment processes

**Figure S2.** Comparison between blood lipid profile and plasma EL

**Figure S3.** The correlation between EL and cognitive impairment in cognitively normal to mild dementia cases

**Figure S4.** The relationship between plasma EL and amyloid pathology or APOE4.

Table S1. Correlation between biomarkers and plasma EL

| Correlation between EL and: | Pearson’s r | *p-value* |
| --- | --- | --- |
| Total Protein | 0.12 | 0.191 |
| Albumin | 0.06 | 0.512 |
| Total bilirubin | 0.07 | 0.446 |
| ALP | 0.02 | 0.868 |
| AST(SGOT) | 0.04 | 0.651 |
| LDH | 0.03 | 0.774 |
| Creatinine | 0.17 | 0.083 |
| Glucose(s) | -0.02 | 0.859 |
| RBC | 0.10 | 0.315 |
| Hb | 0.09 | 0.346 |
| **WBC** | **0.22** | **0.020** |
| **Platelet** | **0.28** | **0.003** |

Abbreviation: ALP, alkaline phosphatase; AST, aspartate aminotransferase; LDH, lactate dehydrogenase; RBC, red blood cell; Hb, hemoglobin; WBC, white blood cell

Table S2. Between-CDR-groups comparisons for EL and HDL-C concentrations.

|  | CDR | | | | |  |
| --- | --- | --- | --- | --- | --- | --- |
|  | 0 | 0.5 | 1 | 2 | 3 | *p* – value |
| N | 12 | 79 | 9 | 7 | 2 |  |
| EL (ng/ml) | 19.4 ± 6.6 | 21.5 ± 9.5 | 30.5 ± 15.3 | 15.5 ± 10.3 | 16.3 ± 2.5 | 0.03 |
| HDL-C  (mg/dL) | 53.9 ± 19.2 | 54.4 ± 15.3 | 48.0 ± 24.2 | 56.3 ± 18.8 | 56.5 ± 6.4 | 0.85 |

| The continuous value is represented by mean ± SD |  |  |  |
| --- | --- | --- | --- |
| *p*-values are for the analysis of variance (ANOVA), | | | |


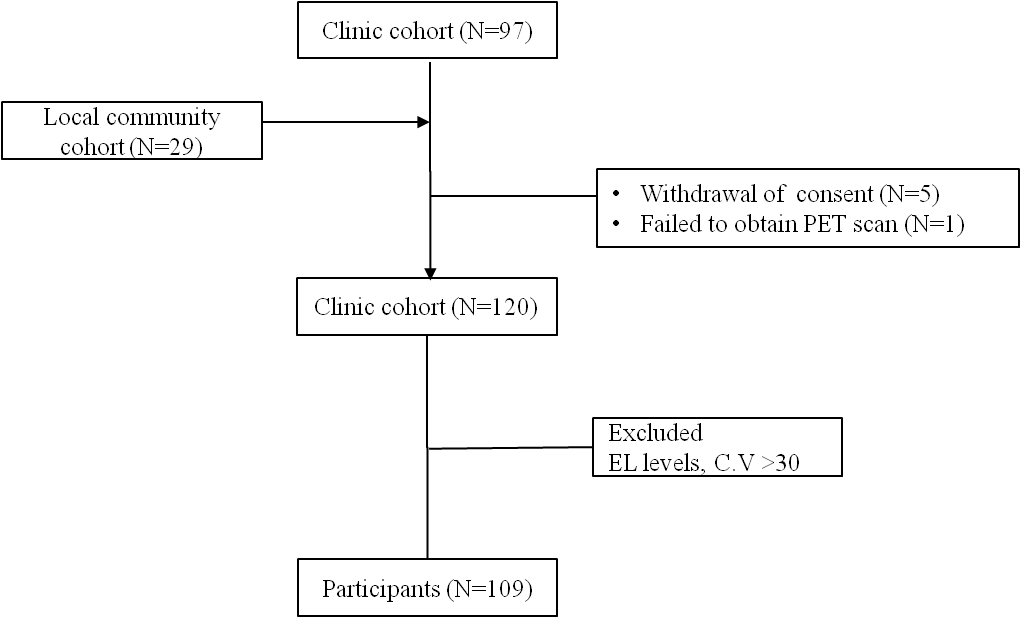


Figure S1. Flow chart of the recruitment processes. A total of 109 participants ≥ 65 years of age were recruited from a clinic and a local community cohort.


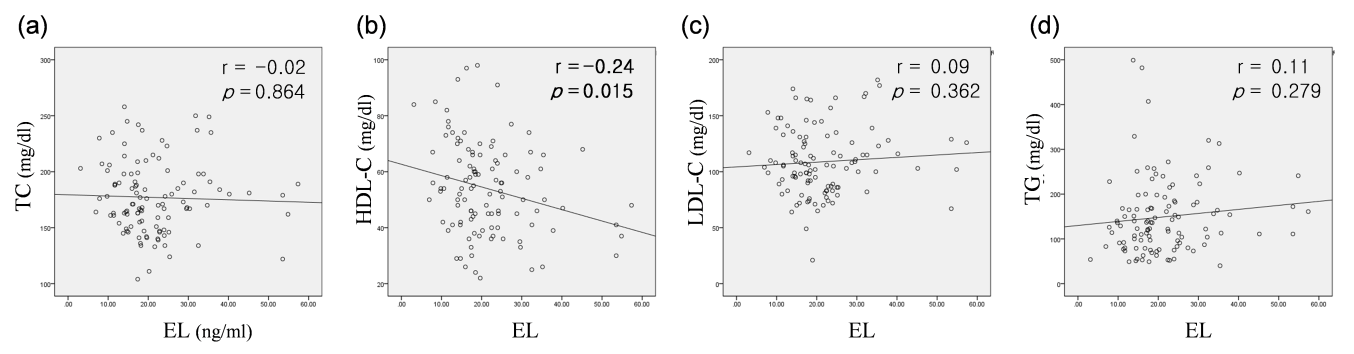


Figure S2. Comparison between blood lipid profile and plasma EL. Correlations for plasma EL levels against total cholesterol (a), HDL-C (b), LDL-C (c) or triglyceride (d) were plotted with Pearson’s r (r) and p-value (*p*), adjusted for age and sex using the partial correlation analysis. Lines show the linear regression curves.


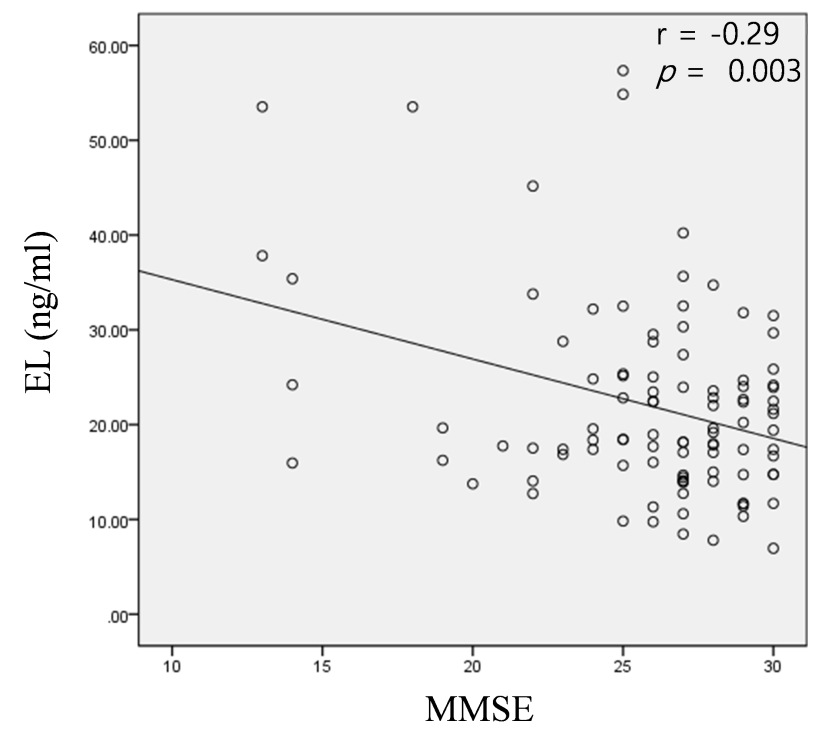


Figure S3. The correlation between EL and cognitive impairment in cognitively normal to mild dementia cases. Correlation coefficient r and p-value between EL and MMSE scores were adjusted for age and sex using the partial correlation test. Lines show the linear regression curve.


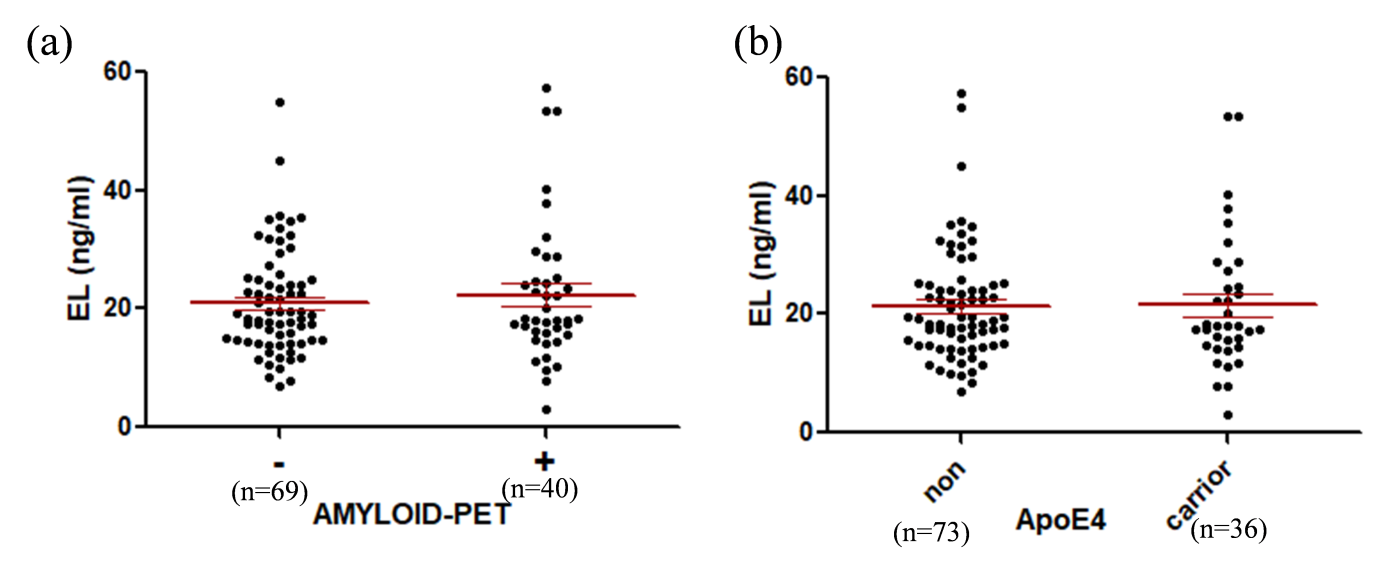


Figure S4. The relationship between plasma EL and amyloid pathology or APOE4. Grouped scatter plots show the plasma EL concentrations according to the amyloid-PET imaging (a) and APOE4 status (b). Red bars represent the mean ± S.E.M of each group.
